# A narrative review of the effect of parent–child shared reading in preterm infants

**DOI:** 10.3389/fped.2022.860391

**Published:** 2022-09-12

**Authors:** Laure Boissel, Jean-Marc Guilé, Sylvie Viaux-Savelon, Charlotte Mariana, Pascal Corde, Fabrice Wallois, Xavier Benarous

**Affiliations:** ^1^Department of Child and Adolescent Psychopathology, Amiens University Hospital, Amiens, France; ^2^INSERM Unit U1105 Research Group for Analysis of the Multimodal Cerebral Function, University of Picardy Jules Verne, Amiens, France; ^3^Pôle de psychiatrie de l'enfant et de l'adolescent, Etablissement Publique de Santé Mentale de la Somme, Amiens, France; ^4^Hospices civils de Lyon, Hôpital de la Croix Rousse, Université Lyon 1, Lyon, France; ^5^Department of Pediatric Neurophysiology, Amiens University Hospital, Amiens, France

**Keywords:** early interventions, parent–infant intervention, neonatal intensive care unit, prematurity, reading

## Abstract

The benefits of book-reading interventions on language development in full-term infants have been well investigated. Because children born preterm face a greater risk of cognitive, language and emotional impairments, this narrative review examines the theoretical evidence, empirical findings, and practical challenges for introducing such intervention to this population. The effect of shared book interventions on typically developing infants is mediated by three components: a linguistic aspect (i.e., exposure to enriched linguistic input), an interactive aspect (i.e., eliciting more synchronous and contingent communication), and a parental aspect (i.e., reducing parental stress and increasing sense of control). Parental shared book reading in a neonatal intensive care unit (NICU) was found to be feasible and well accepted. It provides concrete support for positive parenting in a highly stressful context. Preliminary evidence supports a positive effect of shared reading sessions in physiological parameters of preterm infants in NICU. One study showed that parental shared book reading in an NICU is associated with lower decline in language development during the first 24 months compared to a historical control group. Findings from a community-based birth cohort confirm the positive effect of this intervention on cognitive development with a 2-year-follow up. More structured clinical trials are now needed to confirm these preliminary findings. Questions remain about possible moderators of these interventions, in particular cultural features.

## Effects of reading interventions on infants

Shared book reading is a well-recognized facilitator for language development and reading achievement in preschoolers (1). The quantity of parent–child book reading between 1 and 2.5 years of age specifically predicts children's later receptive vocabulary, reading comprehension, and internal motivation to read (2). The American Academy of Pediatrics issued a policy statement recommending parent–child home reading beginning in early childhood (3). Over the last decades several public-health interventions were developed to encourage parents to do so, both in general populations and in low-income families (e.g., *the Reach Out and Read program, the Bedside Reading program*) (4, 5).

Empirical evidence from longitudinal and intervention studies suggests that reading to preverbal children may be at least as important for long-term child outcomes. Farrant and Zubrick (6) used data from *The Longitudinal Study of Australian Children* to assess the effect of early storybook reading during the first year to later socio-cognitive development. The authors found that the effect of maternal education on children's vocabulary development at 34 months of age was completely mediated by the level of parent–child book reading. Brown et al. (7) compared the effectiveness of a high- and a low-intensity of shared reading interventions for 32 parent–child dyads with typically developing babies 3- to 12-months-of-age (high vs. low-intensity). Increased performance in the high-intensity group was observed for language scores and social communication scores immediately post-intervention and at 2-years-of-age. On this ground, recommendations for the parents to read aloud to their children has been extended to those as young as 2 weeks (3, 8). Other programs were developed to be provided by nurses beginning at childbirth (e.g., “*Let's Read*”, “*Better Beginnings*”) (9, 10).

## Mechanisms involved in the effectiveness of shared reading interventions

Several studies have explored the mechanisms underpinning the effectiveness of shared parental reading on typically developing children.

The exposition of children to enriched language, through exposition to more grammatically enriched linguistic input, is regarded as a central point for the effectiveness of parental reading interventions (2). Weisleder and Fernald (11) demonstrated that the processing speed is affected by the amount of language a child hears, ultimately affecting the acquisition of vocabulary later. In addition, the language used in books is far richer than the language of everyday speech (12). Noble et al. (13) showed that the child-directed speech generated by shared book reading contains significantly more grammatically rich constructions than child-directed speech generated by toy play.

Non-linguistic components of the shared reading sessions could also play an important role in the effectiveness of these interventions. Shared parent–infant reading sessions tend to elicit more interactive communication between infant and caregiver including more frequent verbal responses, eye-contact, touch and feel (14). In this, shared book reading could provide a context for caregivers to provide affective support and enriched communication (15). The meta-analysis conducted by Xie et al. (16) found that shared reading interventions are associated with increased parent–child relationships.

To discriminate the effect of language exposure from other aspects of the communication some studies compared the effect of straight reading to responsive/interactive reading. Most of these reports showed that live reading are more beneficial for children in increasing their language abilities compared to recorded reading (17, 18), while another study found no significant difference (19).

Guide for reading sessions often includes parental advice of paying particular attention to infant social cues. Warm and sensitive parental interactions during the reading sessions are regarded as an important aspect to explain the potential benefit of parent–infant reading sessions. A randomized controlled trial demonstrated that after receiving an 8-week shared reading intervention, parents showed increased sensitivity, elaboration, and reciprocity during shared reading interactions with their infants compared with the control group (20).

In addition to infant-parent interactions, reduced parental anxiety is regarded as a significant mediator of the effect of reading interventions. A. Weisleder et al. (21) conducted a secondary analysis of data from a randomized controlled trial comparing the effect of interactive parental guidance based on play or shared reading. The authors found that decreased parental stress was an independent mediator for the effect of shared reading sessions on behavioral outcomes at 36 months. Interestingly, several longitudinal studies have also supported a link between less parenting stress and increased frequency and quality of shared reading (22–24). Other authors have stressed the importance of considering other parental characteristics, such as harsh parenting that is negatively associated with early shared reading (25).

## General principles in premature infants

Prematurity is a well-known risk factor for neurocognitive impairments. Half of premature infants would have mild-to-moderate cognitive impairments, including language and related learning disorders (26), and one out of five would suffer from severe disabilities (27). Associated medical conditions (e.g., cerebral palsy, sensory impairment) contribute to the observed increased risk for cognitive and language impairment among these children (28).

Prematurity is also a medical context associated with an increased risk of attachment issue (29). The birth of a premature infant is generally associated with a constellation of risk factors contributing to less synchronous and contingent caregiver-infant interactions. These factors can be roughly defined as predominantly involving (i) the early infant's aptitude of communicate, (ii) the parental aptitudes to provide well-adapted interactions based on infant social cues, (iii) and the environmental context providing less opportunity for repeated interactive patterns and ill-adapted sensory stimulation for the developmental age (30, 31). In practice such risk factors co-occur and influence mutually increasing parents' difficulties to connect with their child and vice-versa. Guidelines of good practices for preterm infant care in neonatal intensive care units (NICU) highlight the importance of promoting a high level of parental involvement to reduce the risk of insecure attachment. This involves programs aiming to reduce parental stress, to improve parental sensitivity to infant social signals and to promote attuned parenting.

Considering the importance of early sound exposure to cerebral development and the neural immaturity of preterm infants, much attention has been devoted to infants' sound environment in the days following childbirth (32–34). The brain's structural connectivity and auditory function of premature infants are known to be affected by the exposure to parental speech (35, 36). Compared to a home setting, the NICU is an environment with less opportunity of language exposure with frequent unpredictable sounds (37). Some have wondered whether a deprivation of exposure to parental voice and of caregivers-to-infants communication in an NICE could alter neurodevelopmental and emotional outcomes. Caskey et al. (32) found a positive relation between the amounts of language exposure using a Language Environment Analysis (LENA) device in an NICU, and improvements in neurodevelopmental language testing at 7 and 18 months among those infants.

Early interventions, such as shared reading interventions, that promote both exposure to parental voice and the opportunity of richer parents-infant interactions are therefore worth considering in preterm infants to improve long-term neurodevelopmental and emotional outcomes (38).

## Empirical evidence supporting the effect of storybook reading in preterm infants

The principle of shared book reading sessions in an NICU was presented in a seminal article by Jones and Englestad (39). More recently, two articles described the implementation of shared book reading interventions in an NICU in Italy (40) and in the USA (41) (Table 1). Of note, parent–infant shared reading programs described here do not involve interventions provided by dedicated psychotherapists.

**Table 1:** Studies describing shared reading interventions in premature children.

**Authors**	**Design/population**	**Intervention**	**Assessments**	**Main outcomes**	**Comments**
**Interventions in neonatal intensive care unit**					
Jones and Englestad (39) USA	Description of clinical experience	Parental intervention in neonatal ICU without further information	No	No	
Lariviere and Rennick (42) Canada	Pre-post study design *N* = 116 (*n* = 59 vs. *n* = 57 in the historical control group) 61% male Recruited in NICU (mean 37 GW)	Parental intervention in neonatal ICU Book: chosen by parents in bookshelves and then personalized Duration/frequency: a few minutes every day Setting: at the bedside, in the incubator, or while holding the infant	Parental outcomes: - parenting stress: PSI-SF - parent–infant interaction during reading: qualitative analysis of parental verbatim - frequency of reading: PIAS	- Well accepted interventions with increased parental sense of control, of intimacy and of normalcy - Higher frequency of shared reading sessions 3 months after discharge in the intervention vs. the control group (56 vs. 23% reported reading ≥ 3 times/w.)	- Except belonging to an intervention group no other infant-related or family-related variables predicted the frequency of shared reading interventions at 3 months post discharge - Limitations: historical control group
Walker (41) USA	Description of clinical experience	Parental intervention in neonatal ICU without further information	No	No	Detailed description of possible difficulties in implementing shared book reading sessions
Biasini et al. (40) Italy	Pre-post study design *N* = 76 (*n* = 49 vs. *n* = 27 in the historical control group) Preterm infants % male: nr	Parental intervention in neonatal ICU Book: chosen by parents in bookshelves and then personalized Duration/frequency: “*every time they would think possible and useful*” Setting: at the bedside, in the incubator, or while holding the infant, assisted by nurses for the first sessions	Parental outcomes: - frequency of reading at 6–12 months PIAS Developmental skills: Hearing and Language quotient of the GMDS for 0–2 years assessed at 18 months of corrected age by a psychologist	The intervention was associated with higher GMDS in the language subscale 86% of parents enjoyed reading during the stay in NICU, 89% felt it helped them feel closer to their babies Subgroup analysis: parental satisfaction higher in parents of infants with very low birth weight	Parents were encouraged to use motherese prosody and reinforcing emotional expression
Scala et al. (43) USA	Pre-post study design *N* = 18 Preterm infants (23–31 GW) 41% male	Parental intervention in neonatal ICU Book: chosen by parents Duration/frequency: 15–60 min, twice a day Setting: in the incubator *via* Bluetooth speakers Comparison between recorded and live reading	Physiological data: Cardio-respiratory stability (HR, RR, oxygen saturation, apnea, bradycardia events) 3 and 1 h before reading, during the sessions and 1 h after Parental outcomes: no data	Fewer desaturation during parental reading than prior to reading exposure. This effect persisted up to 1 h after reading exposure	Exploratory analyses showed fewer desaturation events in the live vs. the recorded reading sessions and in maternal vs. paternal reading sessions
Neri et al. (44) Italy	Pre-post study design *N* = 100 (*n* = 55 compared *n* = 45 in the historical control group) 56% male Preterm infant with birthweight below 1,500 g without fetopathy and severe neonatal complications	Parental intervention in neonatal ICU Book: colored-picture picture chosen by parents in bookshelves and then personalized Duration/frequency: no data Setting: at the bedside, in the incubator, or while holding the infant	Developmental skills: Assessment at 3, 6, 9, 12, 18, and 24 months of corrected age by a psychologist with the GMDS-R for 0–2 years Parental outcomes: no data	- No difference in language scores of the GMDS-R between the two groups at 24 months - The decreases observed in the language scores of the GMDS-R was reduced in the Reading Group compared to the Control Group.	Only infants with extremely low birth weight
**Other studies**					
Braid and Bernstein (45) USA	Secondary analysis using the ECLS-B *N* = 1,400 Preterm infants (22–36 GW) 51% male	Item of the short form of the HOME Inventory (direct observation and interview with primary caregiver): reading aloud > 2 times a week (Y/N)	Developmental skills Bayley Mental Scale T-score in children aged 2-year Parental outcomes: no data	Reading aloud > 2 times a week is associated with higher cognitive development scores at follow-up	Finding is consistent after adjusting on neonatal features (child's birth weight, gestational age, and sex), parental features (maternal age, primary and home language, and race/ethnicity, and maternal education) Race/ethnicity and maternal education affect the frequency of parents reading
Zuccarini et al. (46) Italy	Subgroup analysis of a non-randomized controlled study *N* = 23 low-risk preterm children with language delay (*n* = 17 intervention, *n* = 6 control) Aged 37 months 63% male without fetopathy and severe neonatal complications	Parental intervention for late talkers aged 2–3 years Book: chosen by parents Duration/frequency: six 2-h sessions and 2 video-feedback sessions during 2 months Setting: home sessions	Developmental skills (6 months after the interventions): MB-CDI, Words and Sentences Complete Form, BSID-III Parental outcomes: no data	Stronger improvement in expressive syntactic skills (stable or emergent complete sentence) in subjects in the intervention group compared to those in the control group. No different in expressive lexical delay.	The intervention is more complex as only shared book reading with parents involving homework and video guidance

BSID-II, Bayley Scales of Infant and Toddler Development Second Edition; BSID-III, Bayley Scales of Infant and Toddler Development Third Edition; MB-CDI, MacArthur Bates Communicative Development Inventories; CRE, oxygen desaturation; ECLS-B, Early Childhood Longitudinal Study-Birth Cohort; GMFS-R, Griffiths Mental Development Scales- Revised; HOME, Home Observation for Measurement of the Environment; PIAS, Parent–Infant Activity Sheet; PSI-SF, Parenting Stress Index, Short Form23.

Lariviere and Rennick (42) described the experiences of parents involved in a shared book reading intervention in an NICU in Canada with 59 infants. The nurses advised the parents to read a few minutes every day from a book previously selected among the ward library and personalized. Authors reported that 86% of parents involved in reading sessions found the activity enjoyable, and 69% mentioned it helped them to feel closer to their baby. Comparing this to a historical control group, parents involved in this activity were twice as many to report reading three or more times a week to their infants 3 months post NICU discharge (56 vs. 23%).

Biasini et al. (47) replicated this finding among 49 Italian preterm infants compared to 27 subjects in the historical control group. The authors found that a large majority of parents enjoyed reading sessions and most of them felt it helped them feel closer to their babies. The intervention was associated with higher Hearing and Language subscore of the GMDS at 18th month of follow-up.

Scala et al. (43) examined the effect of parental shared reading intervention on preterm infant's physiological parameters (*n* = 18 dyads). Parents read a book to infants in their incubator *via* Bluetooth speakers. Oxygen desaturation decreased during parental reading compared to baseline (3 and 1 h before). This effect persisted up to 1 h after reading exposure. Interestingly, exploratory analyses showed that the effect was stronger for live compared to previously recorded reading sessions and for maternal compared to paternal sessions.

Neri et al. (44) compared the development of language acquisition during the first 24 months following the birth between preterm infants who had parental shared book reading session in an NICU (*n* = 55) and a historical control group (*n* = 45). Authors found no significant difference in language scores of the Griffiths Mental Development Scales-Revised between the two groups at 24 months. However, the analysis of developmental trajectories based on intermediary scores (at 3, 6, 9, 12, 18 months) showed that the decline in language skill observed among all participants compared to expected scores in general population was significantly reduced in the reading group compared to controls.

Braid and Bernstein (45) used data from a large community-based birth cohort to examine in a sample of preterm children (*N* = 1,400) the association between parental shared book reading and children's cognitive development. The authors found that reading aloud at least three times a week was associated with higher Bayley Mental Scale T-scores in 2-year-old preterm children. The relation remained statistically significant after controlling on covariables related to neonatal and maternal characteristics [*β* = 2.7, 95% (CI 1.1, 4.3), *p* < 0.001].

Zuccarini et al. (46) determined the effectiveness of a speech intervention for late talkers aged 2–3 years. The intervention focused on shared reading sessions by parents at home and involved more technical aspects such interactive guidance based on video feedback. The authors noted a stronger improvement in expressive syntactic skills 6 months later after the intervention in subjects in the intervention group (*n* = 17) compared to those in the control group (*n* = 6). No significant difference was found between the two groups with regards to expressive lexical skills.

## Clinical and research implications

### Setting of the reading interventions

Except the study by Zuccarini et al. (46) involving former premature children with language delay, the programs reviewed were generally comparable. This is not surprising since most were based on preexisting interventions such as the *Reach Out and Read program* or the *Bedside Reading program* (4, 5). Several authors insist on the importance that the book be chosen by the parents and then personalized (42, 44). Walker (41) observed that these initial steps encourage the parents to be involved and that shared reading be included in their family routine. In a family from a different ethnical background, the book should be in the parents' native language. Some authors have questioned the benefit of using books with specific rhythm of the sentences encouraging motherese prosody, without clear evidence supporting it. The frequency and duration of reading sessions were not well structured in the reviewed studies. Although most authors advocate the importance of considering the availability of premature infants in order to select the adapted duration and frequency of the reading sessions.

### When it should be implemented?

No studies have been done to examine the most appropriate developmental window for shared reading interventions, although indirect arguments would support that the earlier the more effective. Firstly, exposure to the mother's voice in the peripartum period is thought to play a role in the maturation of the brain circuits involved in hearing in late pregnancy (35, 48). Research in premature babies has focused on the risk of auditive deprivation in premature babies hospitalized in an NICU (32–34, 36). In this sense, reading interventions by parents would contribute to increasing an infant's exposure to maternal voice thus recreating an auditory environment closer to normal pregnancy. Since 28 gestational week preterm infants can discriminate phoneme and change in human voice (49). Such early linguistic processing is partly underpinned by pre-neural circuitry involving the activity of generators of endogenous oscillations since the 25th week of gestational age (30, 31). Secondly, the opportunity of richer parent–child interaction is regarded as a mediator of the effect of shared reading interventions (14, 15). Helping parents to engage in concrete activities is worth considering since many of them find it difficult to spontaneously engage in the relationship with the infant, especially if the infant is placed in an incubator (41). The increased sense of control, normalcy, and intimacy among parents of premature babies after shared reading sessions reported by Lariviere and Rennick (42) is important, as these factors are associated with a lower level of parental anxiety and higher perceived parenting competence (50). Prior reports showed that parental anxiety and low perceived parenting competence predicted less optimal and sensitive parent-to-infant interactions (51) and poorer child emotional development (52). Returning to attachment theory, early intervention offers a therapeutic opportunity to harmonize parent–infant interactions when relational patterns still remain to be consolidated.

### How long it should last?

Lariviere and Rennick (42) showed that reading habits starting in an NICU tend to persist in the following months after return home. The book personalization surely enables to make the shared book sessions more individualized and then to be included in family rituals. Several studies insisted on the possibility for parents to keep the book after intensive care discharge (42, 44). Maintaining shared reading activities with infants over time is not anecdotal. Initially the intervention has been thought to offer parental guidance for the consolidation of positive parenting in a highly stressful context rather than to provide a targeted intervention (39, 41). A large amount of evidence supports the positive effect of shared reading in preschoolers, whether or not they are medically at risk (1, 2). Braid and Bernstein (45) showed that premature infants whose parents reported more frequent shared reading activities had better cognitive development at 2 years old. Unfortunately, the different areas of cognitive functioning were not detailed in this study. These results are consistent with findings from studies conducted in full-term infants (53). In addition, parental worries and concerns about a premature child are not limited to the acute period of infant hospitalization. A higher level of parental anxiety is usually observed during the first years of life (52). For Walker (41), parents involved in a shared reading program in an NICU may experience a higher sense of parental competence when they continue reading to their child as they perceive this activity as if it was validated by medical authorities compared to other activities.

### Clinical challenges for shared reading interventions

The acceptance of shared reading interventions is likely to be poor if the parents are illiterate or have speech impairments and feel uncomfortable with reading. In this context, some teams suggest using a picture book to comment on (41). While books in other languages may be suitable for families from ethnic minorities, reading books to children is not a common practice in all cultures (54). The intervention will make particularly good sense for the parents who are themselves readers and who are pleased to share this activity with their child. Maintaining a feeling of shared parent-to-infant pleasure during the sessions is worth considering (39, 40). Negative side effects have to be expected if the parents experience the program as an additional strain due to medical requirements (55). This is not clear whether music intervention based on infant passive listening without parental interventions may be an alternative here (56). In addition, some families may already be involved in other early intervention programs such as skin-to-skin care. It is important to consider the best way to articulate these interventions, as illustrated in the study conducted by Carvalho et al. (57) where mothers were invited to speak and to sing to their preterm infants during Kangaroo Care in an NICU. Typically, interventions with preterm infants involve an initial phase of parental guidance on an infant's social signals and how parents can adjust to them (41). This non-specific step could be common before offering more specialized interventions based on parents' preferences.

A related question concerns the mechanisms involved in the effectiveness of shared reading sessions in preterm infants. Some research teams have reported a positive effect of music on cerebral maturation in preterm infants (56), with comparable findings regarding tactile stimulations (58). It is possible that the compensation of sensory deprivation contributes to a significant proportion of the effectiveness of such sensory-based stimulations programs for preterm infant in NICU. This would therefore be important to consider the opposite risk of sensory overload due to developmentally excessive sensory stimulations (40). The developmental period and temperamental feature are likely both important to consider to adapt to infants needs.

### Limitations of the current research

The studies on shared reading programs involving volunteers or nurses were not included here. However, considering the generalization of such bedside reading programs in several hospitals, evidence supporting the pros and cons of each approach would be worth investigating in future. In addition, the literature on mother-baby psychotherapies using storytelling by dedicated therapists were not discussed here. It was considered to be more a structured psychotherapy rather than a supportive parenting approach, while a previous report also reported positive outcomes on parental stress and maternal sensitivity (59). By limiting our research to works published in English we may have overlooked studies reporting experiences in other cultural contexts.

### Implications for future research

So far, empirical evidences are too scarce to answer several key questions about parental shared reading interventions, such as the most appropriate setting (in terms of frequency and duration) and responders features (infant and parents ones). The use of historical control groups in two studies (42, 44) limits the conclusions that can be drawn from them. Future research should prospectively recruit the experimental and the control group. An active control group would be worth including (e.g., other programs promoting good parenting) to discriminate the specific effects of shared reading intervention as mentioned by Weisleder and Fernald (11). Neurodevelopmental markers (e.g., physiological parameters, electrophysiology) could be used to indirectly assess the effect of this intervention on brain maturation of regions involved in processing auditory information. Three types of variables could be collected to better understand the mediators of the effect of shared reading intervention:

- Related to language exposure: frequency/duration of sessions, exposure to language in the NICU environment over the same period (either parental and non-parental)
- Related to parent–infant interactions: video of shared reading sessions for direct measurement of parent–child interactions, parental synchrony and parental sensitivity
- Related to parents: level of parental anxiety, sense of parental competence

Another assessment a few months after the intervention will help to determine whether parents have included shared reading in the family routine. Longitudinal studies in community-based or in clinical samples would provide information on the longer-term impact of shared reading practice, in particular on reducing the higher risk of cognitive, language or emotional problems of premature children.

## Conclusion

Preliminary evidence supports the benefit of shared reading intervention in premature infants to support language acquisition and parental bonding. Such programs are feasible and well-accepted in an NICU. Structured clinical trials are now needed to confirm these preliminary findings. The question also remains about which components of shared reading interventions mediates its effectiveness. Answering this could help to better articulate it to preexisting interventions supporting cognitive stimulation and good parenting in premature infants. It would also help to define the group of patients for whom this may be particularly relevant.

## Author contributions

XB, LB, J-MG, FW, PC, SV-S, and CM: study concept, design, and interpretation of data. XB and LB: acquisition of data and drafting the manuscript. J-MG, FW, PC, SV-S, and CM: critical revision of the manuscript for important intellectual content. All authors: final draft. All authors contributed to the article and approved the submitted version.

## Conflict of interest

The authors declare that the research was conducted in the absence of any commercial or financial relationships that could be construed as a potential conflict of interest.

## Publisher's note

All claims expressed in this article are solely those of the authors and do not necessarily represent those of their affiliated organizations, or those of the publisher, the editors and the reviewers. Any product that may be evaluated in this article, or claim that may be made by its manufacturer, is not guaranteed or endorsed by the publisher.
